# Annotations

**Published:** 1888-07-07

**Authors:** 


### ANNOTATIONS.

**Summer Under-clothing.**

FOR persons of civilised habits, whose life is spent in variable climates like our own, the question of underclothing is one of considerable importance. That the body should be maintained in a condition of normal warmth, that perspiration should be unchecked but not too much stimulated, that the feeling of comfort should be complete and permanent—these are desiderata not to be treated with contempt. In winter the underclothing question almost solves itself. Woollen of suitable texture from head to foot is usually all that is required ; but the case is different in summer. For some people, even in summer, thin woollen may be most comfortable and most healthy ; but to the large majority, who, under a blazing sky, feel with Sydney Smith that they would like to "take off their skin and sit in their bones," the mere suggestion of woollen is almost unendurable. Perhaps in this feeling they may be mistaken ; but whether or not, there is no doubt that the feeling exists and is almost universal. A really suitable material for summer underclothing would be welcomed by many, particularly among those classes, such as cricketers, cyclists, agriculturists, and others, who live much in the open air. The cellular clothing, manufactured by the Cellular Clothing Company, is in theory exactly the kind of thing which those who are oppressed by the heat of summer would most desire. Its meshlike structure permits of the access of air to the surface of the body and of the freest exhalation of perspiration. The heat of the body so raises the temperature of the atmospheric stratum immediately in contact with the skin as, it is said, quite to prevent chills. If this be so, and perspiration is freely exhaled whilst chills are avoided, the value of cellular clothing for summer weather is very great indeed. Whilst it is of service to the public that a medical man should point out the rationale of the use of cellular clothing from a health point of view, no medical opinion is needed as to matters of fact ; and every man may easily prove for himself, by experiment, whether or not the clothing fulfils the promises its manufacturers make on its behalf. For ourselves, we have given it a fairly prolonged testing, and find it cool, comfortable, and conducive to health. It is cheap, it is of British manufacture, and is undoubtedly well worthy of a trial.

**Wealth for Women.**

THE average woman dearly loves money. She would agree with Burns that she does not love it for the sake of "hiding it in a hedge," but she would scoff at the poet's love of it merely for the sake of the independence of feeling it brings. She loves "a train attendant," whatever she may care about "the glorious privilege of being independent," and money is the absolute *sine qua non* of "a train attendant." Women—ordinary women—hate economy and small savings. They like to spend, to spend freely, generously, lavishly, and to see themselves clothed and surrounded with all the magnificence which wealth can so easily produce. Women marry for money with much fewer qualms of conscience than men. A man feels, as Anthony Trollope's tailless fox felt, that it is a mean thing to provide himself with a tail at his wife's expense. He may now and then do it as a matter of necessity, but the duty is unpalatable. Many women nowadays cannot marry at all, either a rich or a poor man ; and some of them, with a good sense worthy of sincere admiration, are taking to earning money on their own account on a large scale. The pittances and the patronage doled out to those women of education who elect to be governesses and clerks, are not for the more courageous of the sex. They see that there is profit in the keeping of a shop, and shops accordingly some of them have elected to keep. It is said that one dame of high degree, whose name may be found in the eerage, tries on bonnets herself in the interests of her customers, and tries them on too with much advantage, both to them and to her own exchequer. The son of a gentleman of distinction, it is reported, is essaying to climb the hill of fortune, if not of fame, as a market gardener. Two or three ladies are said to be seriously contemplating a hen-farm near London ? Why not indeed ? If people want money the best thing they can do is to set themselves by some honest means to earn it. The prejudice against trade and traders, especially shopkeepers, is entirely destitute of foundation in reason. It is a wretched survival from times of ignorance and stupidity. Is has spread from the ranks of the highest until it can reach no lower level. Its ridiculousness is seen to perfection in the youth who scorns to be a carpenter because he wants to be a gentleman, and feels quite sure he has carried his point when he has become a clerk on £30 a-year. If women of position and some means will really help forward the movement that has now commenced, and take up trade bravely when their necessities demand, they will render invaluable service not only to their own private exchequers, but also to that large tribe who would die rather than do what those they look up to refrain from doing. It is only conspicuous examples which will reassure the timid and feeble-minded worshippers of "good form."

**Many Ways of Resting.**

THE Plymouth *Western Morning News* is a little shocked at our suggestion that a day in bed is a good thing for the over-tasked citizen. "THE HOSPITAL contains what can only be described as an invitation to occasional laziness," it says. "It actually recommends a day in bed for weary workers." Our western contemporary is shocked at this, and suggests that there are many ways of spending a really restful day without "enduring the blankets for so long a time as thirty-two hours"; and quotes a gentleman who rested from his daily work by re-arranging the books in his library. From this instance we should infer that the *Western Morning News* recommends the enjoyment of the fact of laziness accompanied by a fiction of industry. Well, we recommended fiction also, but bound in book shape, and perhaps tending less to that deceiving of ourselves which high authority condemns. We do not grudge any man his fashion of resting ; if he likes to mould it into a semblance of activity who shall forbid him, though we think that in that case he can never have been thoroughly tired out, never have been in the condition of the oft-quoted old woman whose ideal of heaven was "to do nothing for ever and ever." It requires such a state of exhaustion and nothing less to make one appreciate to the full the bliss of a day in bed—the forgetting for a brief space of cares, anxieties, activities, duties, responsibilities. To a very large extent change of labour is recreation ; it brings another set of faculties into play, and so tends to restore the lost balance of power ; but this only holds good so long as the fatigue is moderate, and we recommended a day in bed only to those whose labour, normal or abnormal, tends to extreme exhaustion. All the things a man thinks he might have done well if circumstances had not doomed him to a city desk or the mill-round of a profession please him when he is moderately tired; the worse he does them the better they please him, by some strange fantastic power of self-delusion. The amateur photographer, of whom the *Western Morning News* speaks so approvingly, is perhaps the most terrible of all hol by-riders, because he does so much to circulate slanders—falsely called portraits—of the various members of his family ; but the amateur violinist runs him close, and so does he who sings out of tune. Far be it from us to grudge any man his little comforts of this kind ; we would even submit to play his accompaniments and be "taken" in several positions if he desired it ; but all these things imply the possession of still a little nervous energy. When he has none, when the fiddle lies unstrung, and he doesn't even want to stain his fingers with chemicals, then we still recommend him our old prescription—a day in bed.

